# Insights in genetic diversity of German and Italian grape berry moth (*Eupoecilia ambiguella*) populations using novel microsatellite markers

**DOI:** 10.1038/s41598-021-83855-0

**Published:** 2021-02-24

**Authors:** Annette Reineke, Alberto Pozzebon, Olivia Herczynski, Carlo Duso

**Affiliations:** 1 Department of Crop Protection, Geisenheim University, 65366 Geisenheim, Germany; 2 Department of Agronomy, Food, Natural Resources, Animals and the Environment, University of Padova, 35020 Padova, Legnaro Italy

**Keywords:** Ecology, Agroecology, Animal migration, Ecological genetics, Population dynamics, Ecology, Agroecology, Animal migration, Molecular ecology, Population dynamics

## Abstract

The grape berry moth *Eupoecilia ambiguella* (Lepidoptera: Tortricidae) is causing significant damage to grape berries, however, little is known on population genetics of this lepidopteran pest insect, hindered so far by the lack of suitable molecular markers. Here we report on the development of ten microsatellite markers of which six were used to characterise 21 *E. ambiguella* populations obtained from two viticultural regions in Germany and Italy. Moths were sampled during two subsequent generations (flights) in the same vineyard as well as in vineyards surrounded by different landscape types. German and Italian populations were genetically differentiated and a significant isolation by distance was evident. No significant divergence was observed among the populations from first or second flight moths, however, inbreeding was higher in first than in second flight populations. Moreover, inbreeding was influenced by habitat composition and complexity of landscape around vineyards, being positively associated with the percentage of area covered by grapevine. Population genetics of *E. ambiguella* could thus be affected by the presence of alternative host plants in viticultural landscapes, which is important in the light of both insecticide resistance management and sustainable pest management.

## Introduction

Genetic diversity of populations of polyphagous insects is shaped by the availability of host resources and thus by landscape composition and heterogeneity. In agricultural landscapes, natural habitats such as hedges, forests, grasslands or other uncultivated sites may provide alternative host plants, overwintering sites or other refugee niches for herbivorous insects at several key stages of their life cycle^1^. Dispersal of individuals inhabiting either cultivated or natural habitats can result in gene flow between pest populations and can be important for sustainable pest management on the landscape scale. For example, a lack of natural habitats can reduce gene flow in pest populations favouring the development of insecticide resistance or can lower the pest’s exposure to parasitoids and predators. Grapevines are cultivated on 7.5 Mio ha throughout the world^2^, mostly in temperate regions with cool, wet winters, and hot, dry summers. Viticultural landscapes can be characterized by grapevine grown both in monoculture or in patchy smaller vineyards, located in highly structured settings with diverse natural habitats in close vicinity to crop areas^3^. Various fungal diseases and insect pests target grapevines throughout the season, resulting in very high levels of pesticide treatments^4^.

The grape berry moths, *Eupoecilia ambiguella* (Hübner) and *Lobesia botrana* (Denis & Schiffermüller) (Lepidoptera: Tortricidae), both native from the Palearctic region, are recognized as key pests of West-European vineyards since the eighteenth century^5^. Typically, *E. ambiguella* is common in the northernmost regions of Western Europe, whereas *L. botrana* is widespread in warmer regions reflecting their different ecological requirements^6^. *E. ambiguella* has been detected from Sicily to southern Scandinavia even outside grape-growing areas and is reported as dominant species in vineyards located in Germany, France (northern regions), Switzerland, Austria, Hungary, Czech Republic and Slovakia^5–7^. The two species coexist in transition areas and their ratio depends on climatic conditions: cooler and rainier seasons favor the dominance of *E. ambiguella* populations while warmer and drier seasons favor *L. botrana*. This is frequently observed in northern Italy^8^. During the last decades, *L. botrana* distribution shifted northwards and the pest invaded new viticultural areas across Europe, even replacing *E. ambiguella*^9^. This trend is expected to become more severe in the future under climate change scenarios^10,11^. Moreover, *L. botrana* is recognized as a highly invasive pest in Californian as well as in Chilean and Argentinian vineyards^12,13^, with *E. ambiguella* assumed to have the same invasive potential in cooler viticultural areas^14^.

Both grape berry moth species overwinter as pupae, usually under the bark of grapevine plants, and flight of first generation moths takes place at the time of bud burst. For *E. ambiguella*, most of the investigations support that at least two generations are completed in a season^5^. In some European areas like France and Italy as well as in warmer years occurrence of a third generation has been reported^6^. Larvae of both species attack flower clusters and grapes of *Vitis* species, causing serious economic damage due to direct feeding activities on berries and increased risk of fungal infections of injured berries. Moreover, grape berry moths are polyphagous with *E. ambiguella* being able to oviposit and develop on more than 30 plant species belonging to the genera *Silene*,* Clematis*,* Ribes*,* Prunus*,* Rubus, Sorbus*,* Rhamnus*,* Cornus*,* Lonicera*,* Viburnum*,* Euonymus* etc.^7^. Most of these woody plant species are common in European landscapes, in particular in natural or secondary stands as hedges or shrubs or at forest edges. Population genetics of *E. ambiguella* in vineyards could therefore be affected by presence of alternative host plants and thus by landscape complexity, yet, the degree to which *E. ambiguella* existence, distribution and persistence in viticultural landscapes is influenced by alternate hosts is not well understood so far.

Assessment of population structures and dispersal capacities of insect pests requires the availability of molecular markers such as microsatellites (simple sequence repeats, SSRs), which are known to be robust, follow a co-dominant inheritance and usually show a high degree of polymorphism^15^. Here, we report on the development of the first set of SSR markers for *E. ambiguella* using a next generation sequencing technique. We further apply these markers to describe genetic structures and diversities in populations of this insect pest from two different regions in Europe (Germany and Italy). Moths were sampled in the same vineyards during two subsequent generations (flights) as well as in vineyards surrounded by different landscape types. Therefore, we were particularly interested in asking the following questions.

1. Are *E. ambiguella* populations originating from two viticultural regions in Germany and Italy genetically differentiated?
2. Are *E. ambiguella* individuals sampled in the same vineyard but in two subsequent generations genetically differentiated?
3. Are genetic structures of populations sampled in vineyards surrounded by forests or other semi-natural habitats different compared to populations sampled in landscapes dominated by monoculture vineyards?

## Material and methods

### Sampling

*Eupoecilia ambiguella* adults were collected in five vineyards in south-western Germany (wine-growing regions Palatinate and Rhinehesse) and six vineyards in north-eastern Italy (Veneto region) (Table 1). These vineyards were classified into four categories based on landscape elements present in a radius of 500 m around the vineyard and proximity to forests and woody elements, as many perennial woody plant species are alternative host plants of *E. ambiguella*. The categories were as follows: (A) isolated vineyards with less than 10% of the area cultivated with grapevines, fully surrounded by forests and woody elements (ca. 90% of the area) or other semi-natural habitats; (B) vineyards surrounded by other vineyards, but located at the border or nearby forests with 20–30% of the area covered by woody elements; (C) vineyards located in highly structured landscapes with orchards, other crops, small villages or houses and 10–25% of the area covered by small patches of woody elements; (D) vineyards surrounded mainly by other vineyards with less than 30% of the area covered with other crops and no or only few woody elements. As a basis for landscape analysis, satellite images provided by Google Earth were used and landscape elements were digitalized by hand in QGIS 2.18.9. For a picture of respective vineyards and surrounding landscapes within a radius of 500 m see Table S1. Between-population distances ranged from 2 to 80 km for the German and from 1 to 30 km for the Italian populations, respectively, with a distance of 450–550 km between populations of both geographic regions. Adult male moths were collected using pheromone traps. In German vineyards Leistadt and Weisenheim (D-LST and D-WSH; Table 1) also female moths were gathered using a liquid trapping device filled with vinegar and grape juice. Collections were performed in 2015 for the first (overwintering generation) and second adult flights (summer brood), respectively (Table 1), except for German population Wolfsheim (D-WFH; Table 1), where only first flight moths were sampled. The term “population” refers to samples taken from the same vineyard and flight (generation). Individuals were stored in 95% ethanol for shipping and at − 20 °C until DNA extraction.

**Table 1:** Geographic origin and summary statistics for 21 *Eupoecilia ambiguella* populations analysed by mean values over 6 SSR loci including total number of individuals (N) analyzed for first and second flight moths, number of alleles (NA) including standard deviation (SD), observed (H_O_) and expected (H_E_) heterozygosity, significance of Hardy–Weinberg equilibrium (HWE) and multilocus estimates of *F*_IS_.

Code^a^	Country	Place	Altitude (m.a.s.l.)	Coordinates	Landscape category^b^	N	NA (± SD)	H_O_	H_E_	HWE *P value* ^e^	*F*_IS_
D-WFH-1	Germany, Rhinehesse	Wolfsheim/St. Johann	226	49° 51′ 38.35″ N, 8° 1′ 32.76″ E	C	24	5.17 (1.17)	0.41	0.61	< 0.0001	0.317
D-DEX-1	Germany, Rhinehesse	Dexheim	171	49° 50′ 21.56″ N, 8° 19′ 4.70″ E	D	24	5.17 (2.04)	0.31	0.59	< 0.0001	0.457
D-DEX-2						24	4.17 (1.72)	0.41	0.50	0.0002	0.196
D-LST-1	Germany, Palatinate	Leistadt	245	49° 29′ 57.72″ N, 8° 9′ 4.74″ E	B	24^c^	4.67 (1.75)	0.24	0.62	< 0.0001	0.618
D-LST-2						24^c^	4.83 (2.04)	0.24	0.58	< 0.0001	0.573
D-WSH-1	Germany, Palatinate	Weisenheim am Berg	270	49° 30′ 17.25″ N, 8° 8′ 59.13″ E	B	24^d^	4.83 (1.47)	0.15	0.61	< 0.0001	0.758
D-WSH-2						24	5.17 (2.32)	0.37	0.61	< 0.0001	0.368
D-ROH-1	Germany, Palatinate	Rohrbach	157	49° 8′ 50.73″ N, 8° 8′ 4.05″ E	D	12	5.00 (1.41)	0.56	0.66	< 0.0001	0.149
D-ROH-2						24	5.17 (1.72)	0.47	0.60	0.0005	0.209
I-FAR-1	Italy	Farra di Soligo, TV	160	45° 54′ 9.26″ N, 12° 6′ 30.70″ E	C	15	3.50 (1.05)	0.32	0.39	0.0003	0.161
I-FAR-2						24	4.00 (1.67)	0.42	0.47	0.0025	0.082
I-SOL-1	Italy	Soligo, TV	240	45° 55′ 24.64″ N, 12° 8′ 25.52″ E	A	24	4.00 (1.27)	0.36	0.45	< 0.0001	0.208
I-SOL-2						24	4.00 (1.67)	0.38	0.43	0.0003	0.133
I-MUG-1	Italy	Mugnai, BL	310	46° 0′ 52.79″ N, 11° 51′ 33.17″ E	B	15	3.00 (1.55)	0.34	0.39	**0.1378**	0.115
I-MUG-2						17	3.67 (1.86)	0.38	0.46	**0.1332**	0.168
I-REF-1	Italy	Refrontolo, TV	213	45° 56′ 13.38″ N, 12° 11′ 22.55″ E	A	19	4.17 (1.47)	0.40	0.56	0.0004	0.291
I-REF-2						24	4.50 (1.64)	0.36	0.44	**0.0138**	0.171
I-FEL1-1	Italy	S. Pietro di Feletto, TV	270	45° 55′ 51.52″ N, 12° 14′ 25.39″ E	C	24	3.83 (1.72)	0.34	0.38	**0.0130**	0.079
I-FEL1-2						24	3.67 (1.63)	0.27	0.38	0.0006	0.288
I-FEL2-1	Italy	S. Pietro di Feletto, TV	227	45° 55′ 48.52″ N, 12° 14′ 3.25″ E	C	24	4.00 (1.55)	0.38	0.42	**0.1219**	0.106
I-FEL2-2						24	4.33 (1.86)	0.40	0.42	0.0011	0.048

If not indicated otherwise, only adult male moths were included in the analysis.

^a^Two flights of moths were analysed per location and were regarded as two populations (designated as − 1 and − 2, respectively).

^b^Landscape categories are (A) isolated vineyards with less than 10% of the area cultivated with grapevines and ca. 90% of the area covered by forests, woody elements or other semi-natural habitats; (B) vineyards located at the border or nearby forests with 20–30% of the area covered by forests or woody elements; (C) vineyards located in highly structured landscape with orchards, other crops, small villages or houses; 10–25% of the area covered by small patches of woody elements; (D) vineyards surrounded mainly by other vineyards (at least 70% of the area), no or only few woody elements. For details see Table S1.

^c^Half of the individuals were male, the other half female moths.

^d^Only adult female moths included in the analysis.

^e^Populations in Hardy–Weinberg equilibrium (*P* ≥ 0.05; Fisher’s exact test) are shown in bold.

### DNA extraction

Total genomic DNA was extracted from adults by using a CTAB-based method^16^ modified by the addition of an isopropanol precipitation step. DNA concentration was measured spectrophotometrically and DNA preparations were stored at − 20 °C.

### Microsatellite marker generation

Microsatellite marker generation, PCR analysis as well as statistical and population genetic analysis was basically carried out according to our previous study on SSR marker identification in the European grapevine moth *L. botrana*^17^. For initial generation of SSR markers via 454 pyrosequencing, DNA was isolated from the head and thorax of ten adult *E. ambiguella* individuals obtained from a laboratory rearing at Geisenheim University as described above. Individual DNA was pooled in equimolar amounts and a total of 5 µg DNA was subjected to 454 pyrosequencing. Microsatellite identification and 454 pyrosequencing was performed commercially at Ecogenics, CH. In a first step, size-selected fragments from genomic *E. ambiguella* DNA were enriched for SSR content by using magnetic streptavidin beads and biotin-labeled CT and GT repeat oligonucleotides. The SSR-enriched library was then analysed on a Roche 454 platform using the GS FLX Titanium reagents. In total, 5526 reads were obtained, which had an average length of 164 base pairs. Of these, 983 contained a microsatellite insert with a tetra- or a trinucleotide of at least six repeat units or a dinucleotide of at least ten repeat units. Applying a stringent set of criteria in a tailor-made pipeline (property of Ecogenics) based on the Primer 3 core code (available from http://primer3.sourceforge.net/releases.php) design of primers flanking the microsatellite motifs was possible in 283 reads. A set of 20 oligonucleotide primer pairs having the highest probability of being functional were accordingly picked and tested for polymorphism in the present study.

### PCR analysis, primer validation and population genetic analysis

The 20 obtained microsatellite markers were initially classified in polymerase chain reactions (PCR) according to their performance and degree of polymorphism in five different *E. ambiguella* populations, each including four individuals using the conditions described below. 10 markers failed to amplify in most of the individuals, formed extensive stutter peaks or showed an excessive background in this initial screening. From the remaining set (Table 2), 6 SSR markers were chosen and were used for analysis of a total of 462 *E. ambiguella* individuals. Amplifications were carried out in 15 µl reaction volumes containing 40 ng of the DNA template in a Bio-Rad C1000 thermal cycler. To allow a fluorescent labelling of the generated PCR products, three primers were incorporated in the PCR reactions according to the method described by Schuelke^18^: 2 pmol of a SSR-specific forward primer with an universal M13(-21) tail at its 5′-end (5′-TGTAAAACGACGGCCAGT-3′), 5 pmol of an unlabelled SSR-specific reverse primer, and 5 pmol of a fluorescently labelled universal M13(-21) primer, which will incorporate the fluorescent dye into the PCR product^18^. For multiplexing of the reactions for capillary electrophoresis this M13(-21) primer was either labelled with BMN5 (Ea_01, Ea_02), DY-681 (Ea_05, Ea_06) or DY-751 (Ea_11, Ea_15). For markers Ea_01, Ea_02, Ea_05 and Ea_15 cycling conditions were according to the following PCR program using the Phire Hot-Start II DNA Polymerase (Thermo Scientific): initial denaturation and hot-start step at 98 °C for 3 min, followed by 30 cycles of 98 °C for 5 s, 63 °C for 5 s and 72 °C for 10 s, additional eight cycles of 98 °C for 5 s, 53 °C for 5 s and 72 °C for 10 s and a final extension at 72 °C for 10 min. Marker Ea_06 and Ea_11 were amplified using the DreamTaq DNA Polymerase (Thermo Scientific) and the following program: initial denaturation at 94 °C for 5 min, followed by 30 cycles of 94 °C for 30 s, 56 °C for 45 s and 72 °C for 45 s, additional 8 cycles of 94 °C for 30 s, 53 °C for 45 s and 72 °C for 45 s and a final extension at 72 °C for 10 min.

**Table 2:** Microsatellite loci identified via 454 pyrosequencing in the European grape berry moth *Eupoecilia ambiguella*.

Locus	GenBank Acc. No	Primer sequence (5′–3′)^a^	Core motif	Size range (bp)	NA	*F*_IS_	H_o_	H_E_	F(0)
Ea_1	MW020739	F: AAGTAACCAGAGTGCCCGAG R: GGCTCCACCGACAGCATC	GT_(14)_	228–263	7	0.326	0.515	0.803	0.217
Ea_2	MW020740	F: AACACCCTCCACCCAGATAG R: TTTTCTTATTCCGTGGTTTAGAAATAC	CA_(15)_	174–180	6	0.076	0.481	0.540	0.068
Ea_3	MW020741	F: ACACCGAGCTTTTTATGCCC R: TGGAATCTTGGCTGCGTTTC	CA_(16)_	198–222	Nd	Nd	Nd	Nd	Nd
Ea_4	MW020742	F: CCTACGTCGTGCTCTCCG R: TGTGTCTCATCCACTGACCG	CA_(21)_	108–128	Nd	Nd	Nd	Nd	Nd
Ea_5	MW020743	F: TCAAACAGTCCGGGGTTTTAC R: TTACCCCCACTCCCCTGTC	CA_(18)_	108–134	8	0.175	0.328	0.420	0.126
Ea_6	MW020744	F: TCGACGAGTTACCACAGAGC R: TTTGACGCCGACTGTTCATC	TG_(12)_	208–220	12	0.263	0.472	0.715	0.195
Ea_8	MW020745	F: CAAAGGGGCGTGTAGAATAGC R: GAAGTATTTCAATGTGTGTGCG	CA_(18)_	127–139	Nd	Nd	Nd	Nd	Nd
Ea_11	MW020746	F: AGATGCAGCGGGTTTTATGC R: AAGGTAGTTCCTACTCATCTCG	TG_(17)_	194–216	9	0.612	0.183	0.508	0.474
Ea_13	MW020747	F: ACCGAATTCCACACGGAAAG R: CATGAGGGAGGCCTATGTCC	CAT_(8)_	116	Monomorphic	Nd	Nd	Nd	Nd
Ea_15	MW020748	F: TCCCAACATCTCCTACACCG R: AATGTCAAGTATGTTAAGAATAACGTC	CA_(23)_	132–156	3	0.426	0.145	0.257	0.276

Single locus statistics for six markers such as observed size range of alleles, number of alleles (NA), average *F*_IS_ and observed (H_O_) and expected (H_E_) heterozygosity as well as null allele frequency (F0) were averaged over 21 different *E. ambiguella* populations.

*Nd* not determined.

^**a**^The first 18 bp of the forward primers used in the present study are not shown as they correspond to an universal M13(-21) tail used for fluorescently labelling the PCR products during the reactions.

PCR products were analysed for SSR allele size via capillary electrophoresis on a GenomeLab GeXP DNA Genetic Analysis System (Beckman). Reactions were loaded as a multiplex analysis and included PCR products of three different fluorescently labelled primers (products of primers labelled with BMN5 blue, DY-751 black and DY-681 green). A subset of respective samples was genotyped twice to assess genotyping and allele-scoring error. Reactions that failed in the first run were also repeated at least twice. Allele sizes were determined using GenomeLab GeXP Version 10.2 (Beckman) software.

### Statistical and population genetic data analysis

Descriptive statistics such as average number of alleles per locus obtained with the respective microsatellite marker or observed (H_O_) and expected heterozygosities (H_E_) as well as deviations from Hardy–Weinberg equilibrium (HWE) and null allele frequency (F0) at each locus were calculated using Cervus version 3.0.3^19^. Within population summary statistics such as number of alleles, H_O_ and H_E_ was computed with Arlequin version 3.5^20^. In addition, the program Genepop version 4.7.5^21^ was used to estimate the inbreeding coefficient (*F*_IS_) and to test for the presence and frequency of null alleles in the populations. To investigate population differentiation, a global estimate of *F*_ST_ as well as population pairwise measures of *F*_ST_ were estimated and tested for significance using FSTAT version 2.9.3.2^22^, with a Bonferroni correction for multiple comparisons^23^ applied to all *P* values from *F*_ST_ estimates. A correction for a positive bias induced by presence of null alleles in the data set on *F*_ST_ estimates was performed using the program FreeNA^24^. To test for an isolation by distance a Mantel test was performed based on the relationships between genetic differentiation (represented by the linearized F_ST_ transformation F_ST_/(1 − F_ST_)) and the logarithm transformation of the geographic distances (in km) using the ISOLDE option in Genepop version 4.2. An analyses of molecular variance (AMOVA, Arlequin 3.5) was performed to assess hierarchical genetic population structures among populations from (1) two different geographical origins (Germany and Italy), (2) two different generations and (3) the four different landscape types surrounding respective vineyards.

The genetic structure of the *E. ambiguella* populations was further assessed using the Bayesian clustering algorithm implemented in the program Structure version 2.3.3^25^. This method assigns individuals to an initially unknown number of *K* genetic populations if their genotypes indicate that they are admixed, ignoring any prior data on sampling locations. The model used for all Structure analyses was based on an assumption of admixed ancestry and correlated allele frequencies among populations^26^. To estimate the most probable number of populations, initially ten independent runs for each *K* from 1 to 10 were carried out with 10,000 burn-in steps followed by 10,000 MCMC (Markov Chain Monte Carlo) iterations^25^. Subsequently, the method described by Evanno et al.^27^ implemented in the program Structure Harvester^28^ was used to infer the most likely value of *K*. Simulations in the program Structure were then run again for the most likely *K* with a burn-in period of 50,000 steps and 100,000 MCMC iterations.

As inbreeding results from mating of related individuals and can be influenced by the availability of host plants in the immediate vicinity, one can assume that inbreeding is higher in landscapes dominated by monoculture vineyards. We assessed the relationship between the inbreeding coefficient (*F*_IS_) and habitat composition by fitting a general linear mixed model using the PROC MIXED of SAS ver. 9.4. In the model we considered the inbreeding coefficient (*F*_IS_) as dependent variable and the percentage of area covered by vineyards in a 500-m radius scale as continuous predictor; effect was tested with an *F* test (α = 0.05). The model accounted for country of origin and generation as random term. Degree of freedoms were estimated using Kenward–Roger correction^29^. Prior to the analysis data were checked for normal distribution and untransformed data were used.

### Cross-species transferability of isolated SSR markers

Amplification of the 6 SSR markers isolated from *E. ambiguella* and thus conservation of primer sequences was tested in three other lepidopteran species of the family Tortricidae, i.e. codling moth *Cydia pomonella* (Linnaeus), plum fruit moth *Cydia funebrana* (Treitschke) and European grapevine moth *L. botrana*. Two adult individuals per species obtained from laboratory cultures of the respective insect species were used for DNA extraction, PCR amplification and marker screening as described above.

## Results

### SSR marker characteristics

Of the 20 markers analysed in an initial marker screening, ten showed stable and reproducible amplification patterns with distinct peaks present in capillary electrophoresis (Table 2). One marker (Ea_13) was monomorphic in all *E. ambiguella* populations analysed and data were thus not included in the subsequent analysis.

Six polymorphic microsatellite markers with a stable amplification profile were further assessed for their suitability and were used to characterise 21 *E. ambiguella* populations. Across populations, these six markers amplified a total of 45 alleles (mean 7.5 alleles per locus, range 3–12 alleles, Table 2). Observed heterozygosities per locus ranged from 0.15 to 0.52 and were lower to those expected under Hardy–Weinberg equilibrium, indicating a deficiency of heterozygotes in the analysed *E. ambiguella* populations and/or the presence of null alleles. A heterozygous deficit was also indicated by positive *F*_IS_ values obtained for all markers (Table 2). Estimated frequencies of null alleles were variable depending on the respective SSR marker and *E. ambiguella* population (data not shown). Averaged over all 21 populations, null allele frequencies varied between 0.07% (marker Ea_2) and 47% (marker Ea_11) (Table 2).

SSR marker Ea_3 successfully amplified a fragment of 210 bp size in one of the two codling moth (*C. pomonella*) individuals tested (data not shown). For none of the other *E. ambiguella* SSR markers developed in this study an amplification product was detected in individuals of the three other tortricid species tested, indicating that respective primer sequences and repeat motifs are not conserved in members of this family.

### Genetic diversity and structure of *E. ambiguella* populations

Genetic diversity within populations was moderate to high (mean H_E_ = 0.54 across all loci and populations; range 0.38–0.66; Table 1), while the level of inbreeding varied substantially between populations with a range of *F*_IS_ of 0.05–0.76 (Table 1).

For all German populations, observed heterozygosities were different from expected heterozygosities and populations were thus not in Hardy–Weinberg equilibrium, while 5 out of 12 Italian populations were not significantly deviating from Hardy–Weinberg equilibrium (Table 1). Moreover, Italian *E. ambiguella* populations had overall lower *F*_IS_ values than German ones, indicating that inbreeding can be assumed to be insignificant in these populations. Overall, in most German and Italian populations, *F*_IS_ values obtained for second-flight moths were lower than those obtained for first-flight moths in the same vineyard (Table 1).

The overall *F*_ST_ value of 0.064 indicated a low to moderate level of genetic differentiation between the 21 *E. ambiguella* populations included in this study. The 95% confidence interval (CI) excluded zero (0.042–0.088 determined by 1000 bootstraps over all loci) implying a significant amount of genetic differentiation. If null alleles were excluded from the analysis according to the method described by Chapuis and Estoup^24^ and using program FreeNA the global *F*_ST_ value was similar (*F*_ST_ = 0.069, 95% CI 0.049–0.093) suggesting that the presence of null alleles in the dataset had no impact on the accurate estimation of *F*_ST_.

Of the 210 pairwise *F*_ST_ values calculated among the 21 populations, 129 had a 95% CI excluding zero, suggesting a significant genetic differentiation between the respective populations (Table 3). Pairwise *F*_ST_ values were in all cases comparable when the refined estimation method excluding null alleles^24^ was used (data not shown). Except for two population pairs, all German populations significantly differed from all Italian ones with a moderate to pronounced level of differentiation. Genetic differentiation between Italian populations was often not evident and, in most cases, not significant with overall very low *F*_ST_ values).

**Table 3:** Pairwise *F*_ST_ estimates between populations of *Eupoecilia ambiguella* (for details on locations and code see Table 1).

	D-WFH-1	D-DEX-1	D-DEX-2	D-LST-1	D-LST-2	D-WSH-1	D-WSH-2	D-ROH-1	D-ROH-2	I-FAR-1	I-FAR-2	I-SOL-1	I-SOL-2	I-MUG-1	I-MUG-2	I-REF-1	I-REF-2	I-FEL1-1	I-FEL1-2	I-FEL2-1
D-DEX-1	**0.034**																			
D-DEX-2	0.044	**0.052**																		
D-LST-1	− 0.006	0.003	0.028																	
D-LST-2	0.030	**0.037**	0.015	− 0.007																
D-WSH-1	0.013	**0.029**	0.026	− 0.018	− 0.021															
D-WSH-2	0.025	**0.035**	**0.036**	− 0.015	− 0.006	− 0.017														
D-ROH-1	**0.039**	**0.050**	0.037	0.017	0.001	− 0.007	0.021													
D-ROH-2	**0.081**	**0.068**	0.057	**0.043**	0.020	0.012	**0.040**	− 0.005												
I-FAR-1	**0.181**	**0.146**	**0.216**	**0.136**	**0.146**	**0.117**	**0.117**	**0.175**	**0.155**											
I-FAR-2	**0.097**	**0.092**	**0.124**	**0.068**	**0.069**	**0.051**	**0.050**	**0.106**	**0.100**	0.004										
I-SOL-1	**0.136**	**0.132**	**0.163**	**0.098**	**0.093**	**0.076**	**0.079**	**0.127**	**0.122**	0.008	0.000									
I-SOL-2	**0.109**	**0.105**	**0.142**	**0.074**	**0.077**	**0.061**	**0.060**	**0.120**	**0.114**	0.012	**− 0.011**	− 0.007								
I-MUG-1	**0.077**	**0.083**	**0.120**	**0.065**	**0.098**	**0.072**	**0.072**	**0.134**	**0.142**	0.086	**0.039**	0.081	0.042							
I-MUG-2	**0.085**	**0.099**	**0.107**	**0.061**	**0.056**	**0.040**	**0.044**	**0.079**	**0.074**	**0.039**	0.001	0.006	− 0.008	0.027						
I-REF-1	**0.066**	**0.059**	**0.081**	**0.035**	**0.037**	0.024	0.031	**0.038**	**0.051**	**0.051**	0.017	0.025	0.022	0.042	− 0.004					
I-REF-2	**0.111**	**0.111**	**0.158**	**0.073**	**0.083**	**0.059**	**0.063**	**0.123**	**0.121**	0.023	− 0.002	0.000	**− 0.011**	0.047	0.006	0.025				
I-FEL1-1	**0.150**	**0.156**	**0.170**	**0.116**	**0.116**	**0.093**	**0.094**	**0.151**	**0.137**	0.020	0.010	0.006	0.001	0.060	0.006	**0.036**	0.005			
I-FEL1-2	**0.116**	**0.117**	**0.130**	**0.087**	**0.093**	**0.075**	**0.073**	**0.134**	**0.131**	0.028	− 0.001	0.017	− 0.007	0.009	− 0.003	**0.032**	0.005	− 0.006		
I-FEL2-1	**0.133**	**0.152**	**0.175**	**0.098**	**0.095**	**0.073**	**0.076**	**0.143**	**0.135**	**0.029**	0.003	− 0.005	− 0.002	**0.087**	0.017	0.046	− 0.002	0.007	0.019	
I-FEL2-2	**0.130**	**0.127**	**0.165**	**0.087**	**0.095**	**0.071**	**0.069**	**0.146**	**0.137**	0.020	0.002	0.000	**− 0.012**	0.053	**0.011**	0.047	− 0.006	0.004	− 0.001	− 0.005

Bold typeface denotes pairwise *F*_ST_ estimates with a 95% CI that excluded zero implying significant genetic differentiation. A correction for a positive bias induced by presence of null alleles in the data set was performed using the program FreeNA.

**Figure 1:**
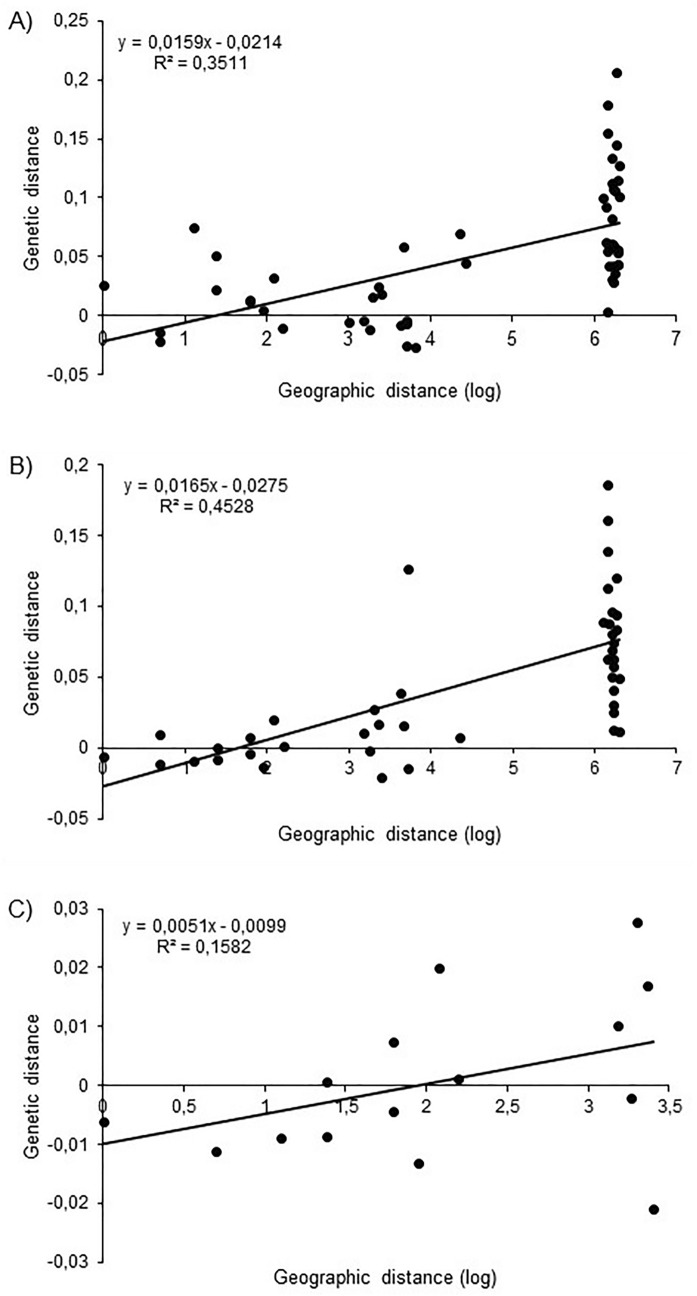
Isolation by distance in *E. ambiguella* populations showing pairwise genetic distances between pairs of populations (F_ST_/(1 − F_ST_)) against logarithm of geographic distance (in km). (**A**) Isolation by distance for first flight populations from Germany and Italy; (**B**) Isolation by distance for second flight populations from Germany and Italy; (**C**) Isolation by distance for second flight populations from Italy.

We found significant relationships between genetic and geographic distances when all populations from both countries and both generations were included in the analysis (first flight Mantel test *P* < 0.0001, Fig. 1a; second flight Mantel test *P* = 0.004, Fig. 1b). When populations were analysed separately for country and generation, respectively, this pattern of isolation by distance was apparent only for pairs of Italian populations sampled during the second flight (Mantel test *P* = 0.004, Fig. 1c).

**Figure 2:**
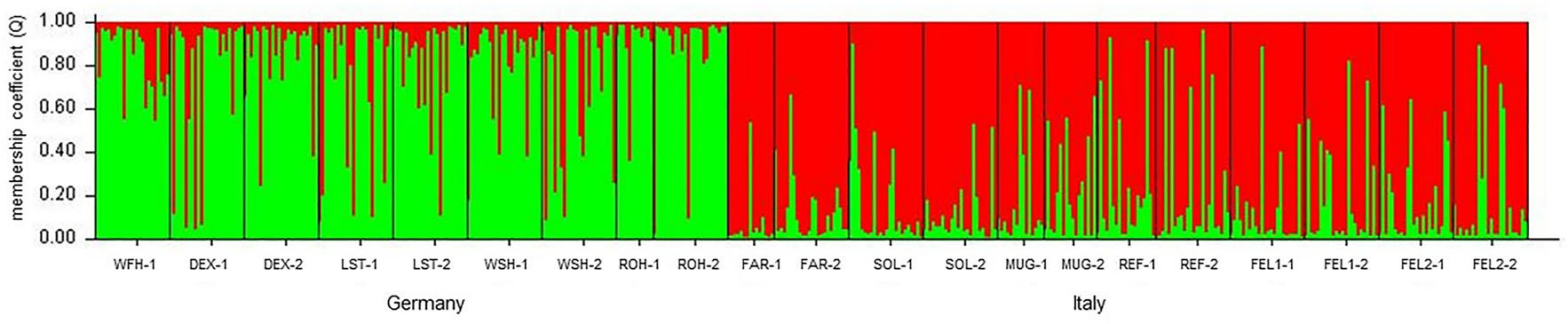
Bayesian assignment of *E. ambiguella* individuals to each of the *K* = 2 identified clusters (green: cluster 1; red: cluster 2). Each bar represents the estimated membership coefficient (Q) for each individual in each cluster. Countries of origin are indicated at the bottom of the graph. For abbreviation and details of collection sites see Table 1.

**Figure 3:**
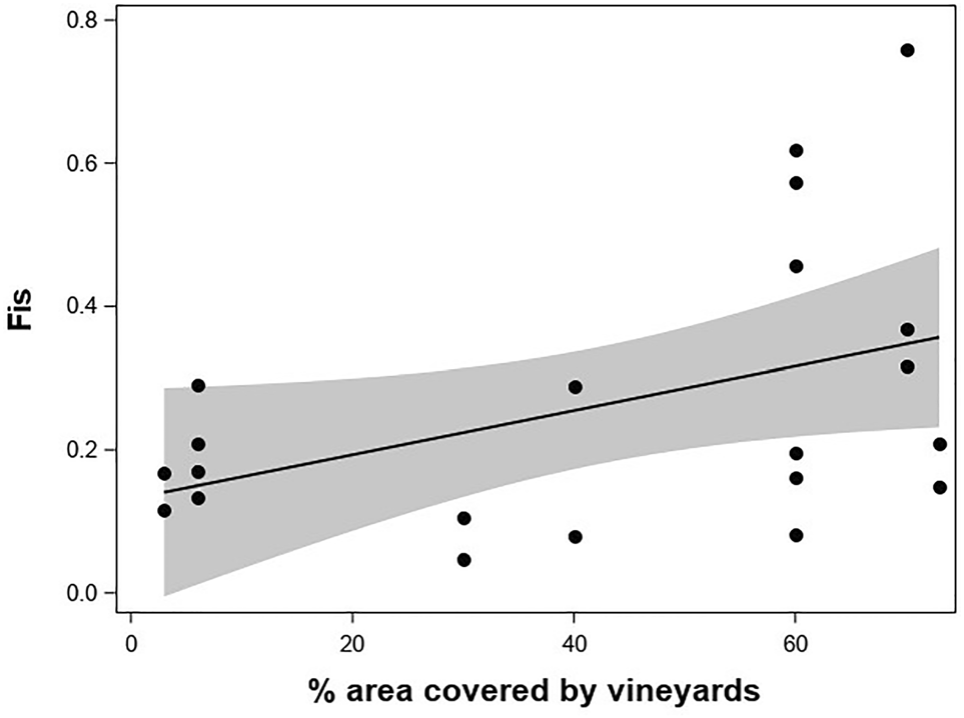
Relationship between percentage of area covered by vineyards (in a 500 m radius) and the inbreeding coefficient (F_*IS*_) in *E. ambiguella* populations. The line represents the prediction for the fixed effect and associated 95% confidence intervals (shaded in gray).

Multiple simulations using software Structure suggested that the *E. ambiguella* samples included in this study represented *K* = 2 genetic clusters, supported by the calculated Δ*K* values^27^ (Fig. 2; Fig. S2). Assignment of *E. ambiguella* individuals to these two genetic groups was generally consistent with the geographic origin of the respective population, as one cluster represented populations from Germany (denoted in green color in Fig. 2), while Italian populations were in most cases assigned to the second cluster (denoted in red color in Fig. 2). The inbreeding coefficient (F_*IS*_) was influenced by habitat composition being positively associated with the percentage of area covered by vineyards (*F*_1,19_ = 4.39; *P* = 0.049; Fig. 3).

To estimate hierarchical genetic structure of *E. ambiguella* populations an AMOVA was performed. Populations were either grouped according to geographic region (Germany, Italy), flights (generation) of moths or landscape type surrounding vineyards. AMOVA indicated weak but significant variance among *E. ambiguella* populations from the two different geographic regions (*F*_CT_ = 0.087, *P* < 0.0001), however almost 89% of the genetic variation was explained by variability within populations (model (1), Table 4). No significant divergence was observed among the populations from first- or second-flight moths (*F*_CT_ = 0.004, *P* = 0.19 for German and *F*_CT_ = − 0.003, *P* = 0.76 for Italian populations) (model (2), Table 4). Finally, AMOVA revealed a weak but significant variance among *E. ambiguella* populations from the four different landscape types (*F*_CT_ = 0.039, *P* < 0.0001), yet in this model 92% of the genetic variation occurred within populations (model (3), Table 4).

**Table 4:** Results of analysis of molecular variance (AMOVA) comparing genetic variation in *E. ambiguella* populations using three different models.

Model	Source of variation	df	Sum of squares	Variance components	% of variation	Fixation indices	*P* value
(1) Country	Among countries	1	68.70	0.148	8.7	0.087 F_CT_	< 0.0001
	Among pops. within countries	19	60.06	0.040	2.34	0.026 F_SC_	< 0.0001
	Within populations	903	1320.05	1.511	88.95	0.111 F_ST_	< 0.0001
	Total	923	1448.82	1.699			
**(2) Moth flights**							
(a) Germany	Among moth flights	1	6.03	0.008	0.43	0.004 F_CT_	0.19
	Among pops. within generations	7	29.05	0.056	3.05	0.031 F_SC_	< 0.0001
	Within populations	399	681.17	1.782	89.52	0.035 F_ST_	< 0.0001
	Total	407	716.25	1.846			
(b) Italy	Among moth flights	1	1.54	− 0.003	− 0.25	− 0.003 F_CT_	0.76
	Among pops. within generations	10	23.44	0.026	1.95	0.019 F_SC_	< 0.0001
	Within populations	504	638.88	1.300	98.30	0.017 F_ST_	< 0.001
	Total	515	663.86	1.323			
(3) Landscape	Among landscape types	3	55.36	0.065	3.93	0.039 F_CT_	< 0.0001
	Among pops. within landscape type	17	73.41	0.067	4.05	0.042 F_SC_	< 0.0001
	Within populations	903	1320.05	1.511	92.01	0.080 F_IT_	< 0.0001
	Total	923	1448.82	1.643			

Model (1) includes populations from 2 different countries (Germany and Italy), Model (2) includes populations from 2 flights calculated separately for German and Italian populations, and model (3) groups populations according to landscape type surrounding respective vineyards (see Table 1).

## Discussion

By using a new set of microsatellite markers this study is the first one to analyse genetic structures in European grape berry moth *E. ambiguella* populations. Results showed a moderate to high degree of diversity between *E. ambiguella* populations sampled in two viticultural regions in Germany and Italy. Moreover, a clear geographic structure was observed in our samples, evident through a significant isolation by distance and through results from Structure analysis. In the later, *E. ambiguella* populations were grouped according to their German or Italian origin in two clearly separated clusters. This indicates a limited genetic exchange between both geographic regions and thus a limited active or passive dispersal of moths from both countries. Active dispersal via flight of moths is unlikely to occur as adult grape berry moths have a relatively low active dispersal pattern and do not migrate over substantial distances^30–32^, however, human-aided dispersal resulting for example from the global trade of plant material could be one way for displacement of e.g. pupae overwintering on the grapevine plant.

All *E. ambiguella* populations included in this study had positive F_IS_ values indicating a deficiency of heterozygotes. This might be due either to the presence of nonamplifying (or null) alleles which is frequently observed in Lepidoptera including the closely related species *L. botrana*^17^. Alternatively, a sampling bias might have caused the apparent heterozygote deficiency, producing a Wahlund effect^33^. Sampling via pheromone-trapping of adults might have actually sampled a pool of individuals from the respective vineyard and/or from subpopulations established on alternative host plants in close proximity. Thus we might in fact have sampled a mix of two or more genetically different subpopulations. A Wahlund effect and null alleles both increase F_IS_ values and produce apparent heterozygote deficits.

The comparison between adults belonging to the first and second flights facilitated additional insights on factors affecting genetic structures of grape berry moth populations. In both German and Italian populations, inbreeding was higher in the first than in the second flight. We can assume that adults caught in pheromone traps in spring originated from overwintered pupae, thus from larvae which developed in these sites in the previous summer and fall. Adults collected during the second flight could include individuals coming from different vineyards and host plants occurring in the surrounded areas. Based on these results, we can speculate that dispersal among different vineyards and alternative host plants might be influenced by seasonal parameters and is apparently higher in the second flight than in the first one. An increase in population densities as well as higher temperatures during summer can promote the flight activity of *E. ambiguella,* and this can be one of the explanations for the differences observed here. A different dispersal capacity between the two *E. ambiguella* flights has implications on its management. First generation larvae infest flowers, while larvae of the second generation damage berries, causing weight loss and reduced berry quality. Accordingly, economic injury levels adopted for the second generation are much lower than those for the first one, with control intervention being unnecessary in most of the cases^34^; however, one can argue that intervention against the first generation brood can have consequences on the extent of second generation infestation. Data on inbreeding coefficients provided here suggest that grape berry moth populations of the first (derived from the first flight adults) and second (derived from the second flight adults) generation are not strictly related, with the first likely derived from adults which developed in the same vineyards, while the second originated from individuals coming also from surrounding areas. Consequently, the control of the first generation infestation cannot be justified as a preventive measure for the control of the second generation.

A lower level of inbreeding was found in Italian compared to German populations, which might again be due to a Wahlund effect and/or the presence of null alleles as discussed above. Moreover, most of the German populations were collected in vineyards mainly surrounded by other vineyards, thus in intensively cultivated areas, while Italian populations were obtained from vineyards located in landscapes with a higher complexity and percentage of non-vineyard habitats (i.e., semi-natural, urban and other crops areas). Habitat composition at 500 m’ radius scale, as considered in the present study, thus seems to affect the population structure of *E. ambiguella* populations. Flight distances for grape berry moths are usually less than 100 m^30–32^ and adult moths tend to mate with individuals nearby in the vineyard right after emergence from pupae. Thus, a certain degree of isolation, with segregation into metapopulations in different vineyards, is likely to occur in diverse and fragmented landscapes which offer other host plants beside grapevine. This is supported by results of hierarchical genetic structure analysis of *E. ambiguella* populations. AMOVA indicated a weak but significant genetic variation among *E. ambiguella* populations sampled in vineyards surrounded by landscape types of different complexity and with different percentages of areas covered by grapevine plants. Insights in the effects of landscape features on genetic composition of pest insect populations is however rare so far. Codling moth (*C. pomonella*) populations in Chile showed a significant but weak genetic differentiation between adults collected in managed and unmanaged orchards, indicating active adult movement between both habitat types^35^. For melon aphids (*Aphis gossypii* Glover) it has been shown that genetic diversity of aphid populations on watermelons cultivated in greenhouses was significantly associated with certain landscape elements in two counties in China^36^. In contrast, a limited gene flow between grain aphid (*Sitobion avenae* Fabricius) populations colonizing cereal crops as well as uncultivated hosts growing in field margins was detected in an area in Western France^37^. Overall, research on interactions between landscape features and evolutionary processes such as gene flow and selection, a research topic referred to as landscape genetics^38^, is becoming increasingly important for understanding dispersal capability of pest insects as well as their natural enemies. In this regard, a recent review by Tscharntke et al.^39^ point to the fact that natural habitats surrounding agricultural fields can be a major source of pests if they provide an environment that is of greater benefit for pest insects than for natural enemies. Understanding the relevance of landscape composition on genetic structures of pest populations is also important in the light of insecticide resistance management and for the deployment of sustainable pest management strategies taking ecosystem services of landscape elements into account. The availability of molecular markers, like the SSR markers developed in this study, are a prerequisite for future studies on insect landscape genetics.

## Supplementary Information

Supplementary Information.
