# Supplementary material for: Insights in genetic diversity of German and Italian grape berry moth (Eupoecilia ambiguella) populations using novel microsatellite markers
Source: Sci Rep. 2021 Feb 24;11:4485. doi: 10.1038/s41598-021-83855-0 (PMC7904833; doi:10.1038/s41598-021-83855-0)
Supplement: Supplementary file 1 — Supplementary Information. [file 41598_2021_83855_MOESM1_ESM.pdf]

Insights in genetic diversity of German and Italian grape berry moth (*Eupoecilia ambiguella)* populations using novel microsatellite markers

Annette Reineke[1*], Alberto Pozzebon[2], Olivia Herczynski[1], Carlo Duso[2]

[1]*Geisenheim University, Department of Crop Protection, D-65366 Geisenheim, Germany*

[2]*University of Padova, Department of Agronomy, Food, Natural Resources, Animals and the Environment, 35020 Legnaro (Padova), Italy*

S1 Table. Classification of landscape surrounding vineyards used for sampling *E. ambiguella.* The circle represents a radius of 500 m around respective vineyards, scale is 1:7500. For details on vineyards see Table 1.

**A Isolated vineyards with less than 10% of the area cultivated with grapevines; vineyards surrounded by forests or woody elements (ca. 90% of the area)**	
I-REF: Italy, Refrontolo	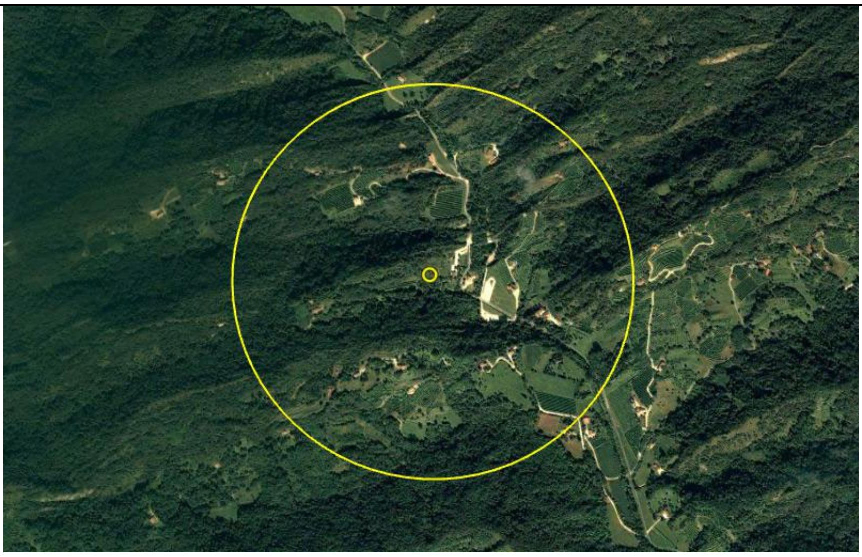
I-SOL: Italy, Soligo	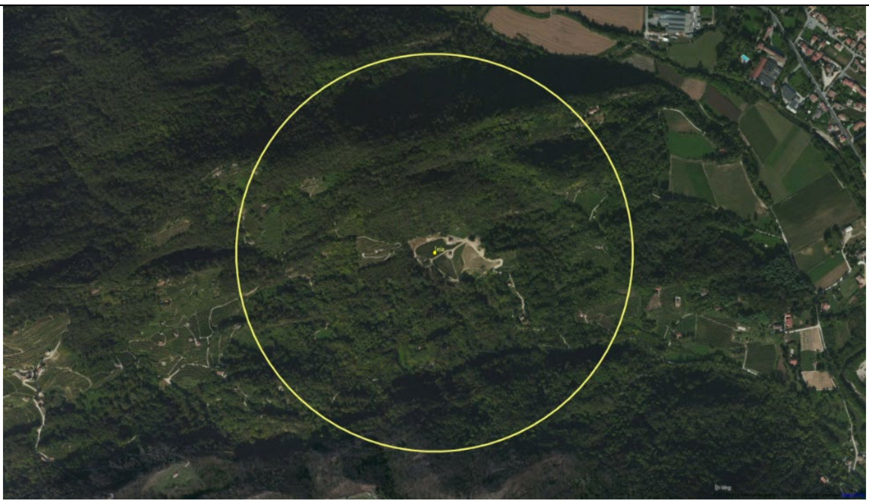

**B Vineyards located at the border or nearby a forest; 20-30% of the area covered by forests or woody elements**	
D-WSH: Germany, Weisenheim	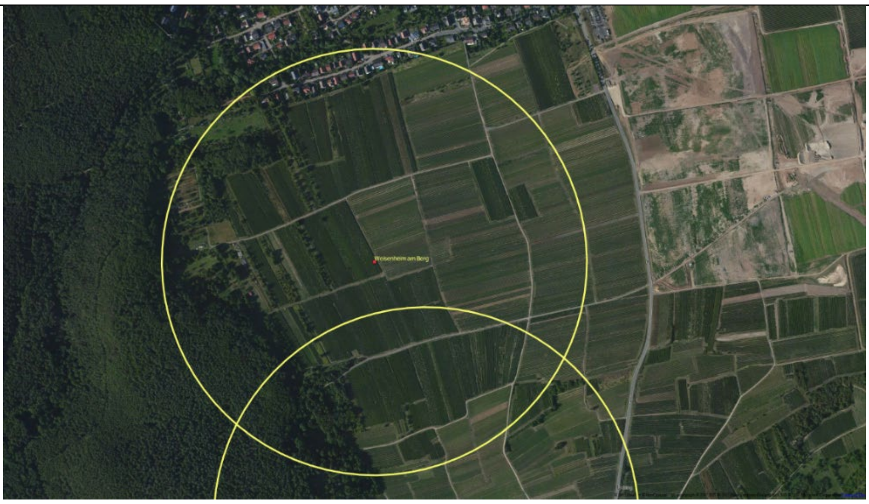
D-LST: Germany Leistadt	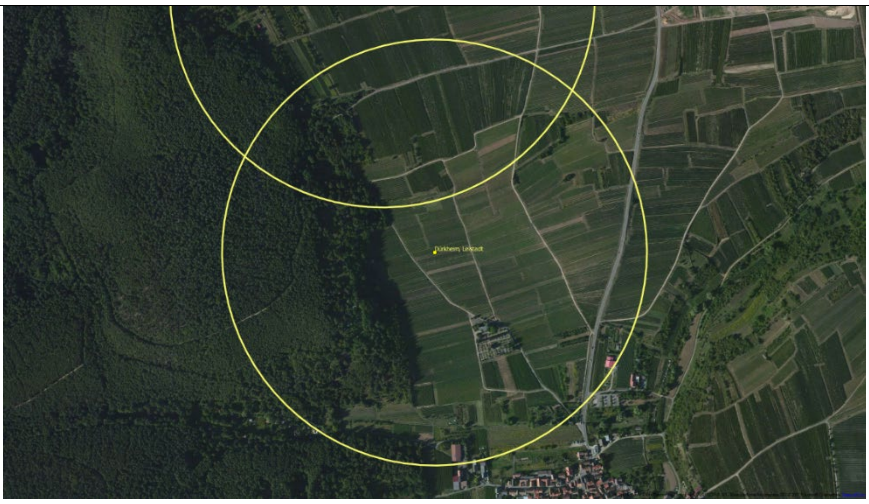
I-MUG: Italy, Mugnai	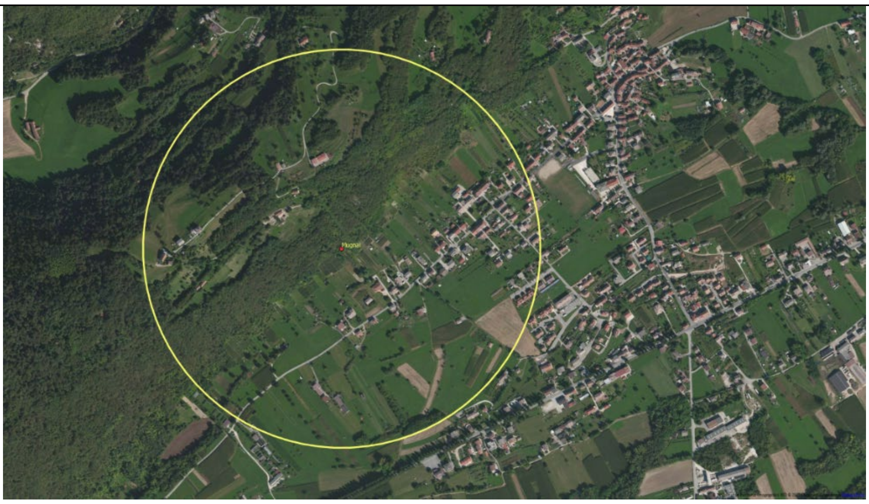

C Vineyards located in highly structured landscape with orchards, other crops, small villages or houses; 10-25% of the area covered by small patches of woody elements	
D-WFH: Germany, Wolfsheim	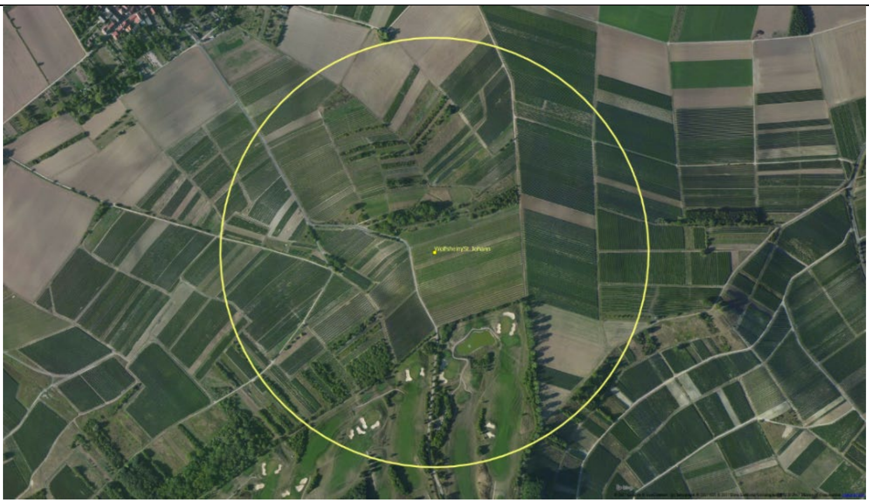
I-FAR: Italy, Farra di Soligo	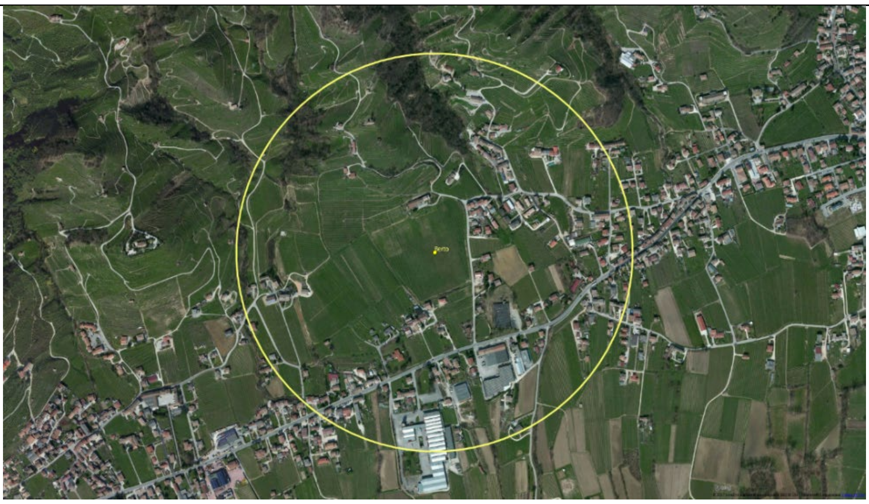
I-FEL1: Italy, S. Pietro di Feletto;<br><br>I-FEL2: Italy, S. Pietro di Feletto	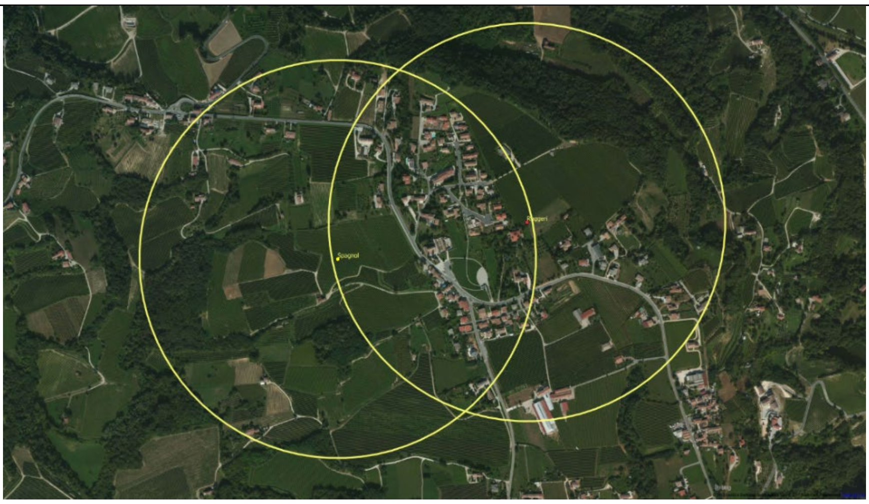

**D Vineyards surrounded mainly by other vineyards (at least 70% of the area); less than 30% of the area covered with other crops; no or only few woody elements**	
D-ROH:<br>Germany,<br>Rohrbach	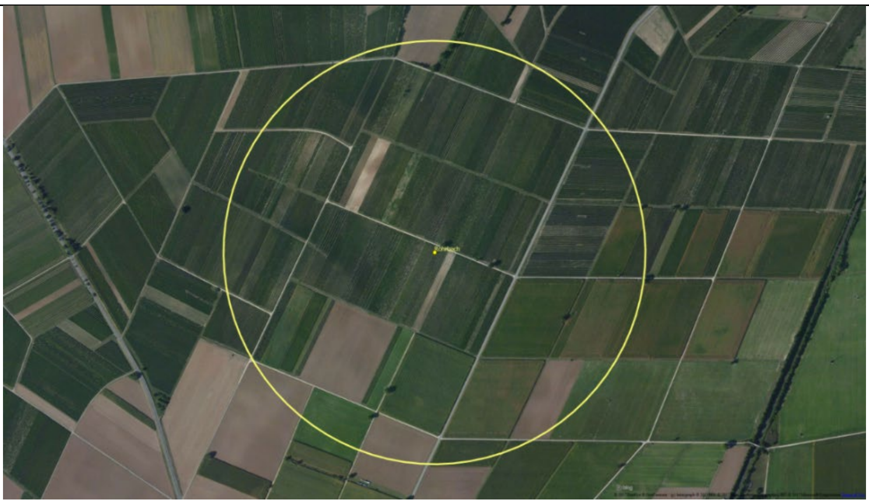
D-DEX:<br>Germany,<br>Dexheim	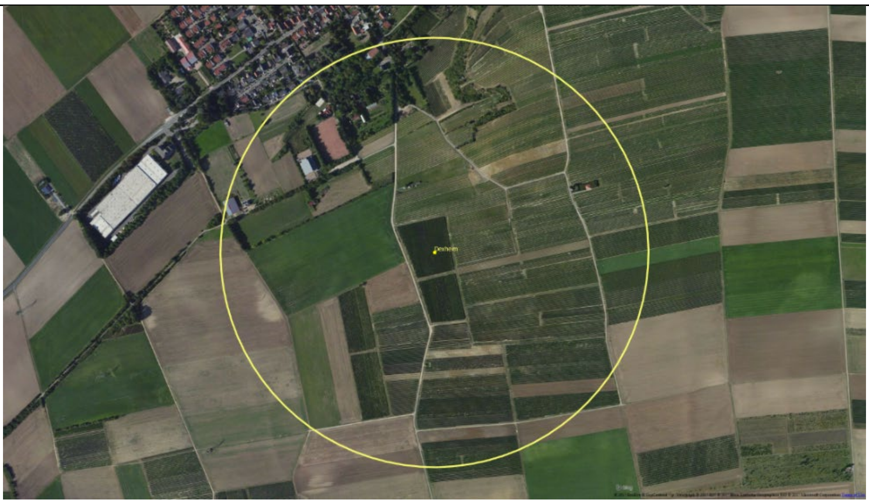

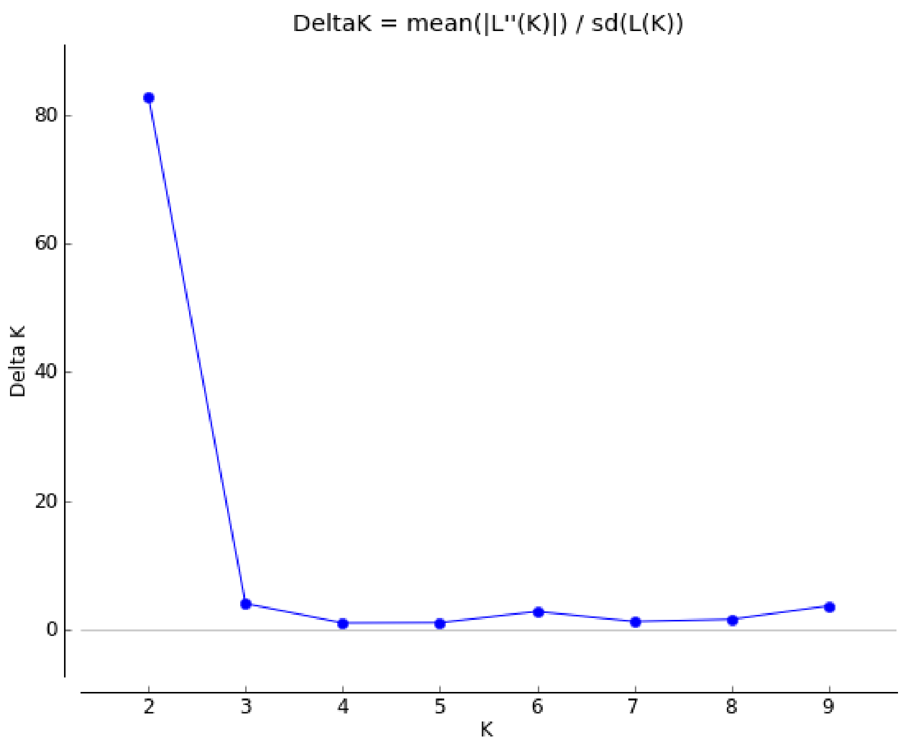

S2 Figure. Inference of the optimal number of genetic clusters *of E. ambiguella* populations calculated by the method of Evanno et al. [26] . *E. ambiguella* samples included in this study represented $K = 2$ genetic clusters, supported by the calculated ΔK values.
